# Lack of Adjuvant Radiotherapy May Increase Risk of Retropharyngeal Node Recurrence in Patients with Squamous Cell Carcinoma of the Head and Neck after Transoral Robotic Surgery

**DOI:** 10.1155/2013/727904

**Published:** 2013-06-13

**Authors:** Waleed F. Mourad, Dukagjin M. Blakaj, Rafi Kabarriti, Rebekah Young, Rania A. Shourbaji, Jonathan Glanzman, Shyamal Patel, Ravindra Yaparpalvi, Shalom Kalnicki, Madhur K. Garg

**Affiliations:** ^1^Department of Radiation Oncology, Montefiore Medical Center, Albert Einstein College of Medicine, New York, NY 10461, USA; ^2^Department of Radiation Oncology, Beth Israel Medical Center, New York, NY 10003, USA

## Abstract

*Purpose*. Transoral robotic surgery (TORS) has increased in popularity in the management of squamous cell carcinoma of the head and neck. However, TORS does not address the neck or retropharyngeal nodes (RPN). In the current report, we highlight the impact of the lack of adjuvant radiotherapy on RPN recurrence after TORS. *Materials and Methods*. A 58-year-old Caucasian male presented with squamous cell carcinoma of the head and neck of unknown primary. He was offered radiotherapy as a definitive management for clinical stage T0N2aM0, stage IVA, but he opted to left neck dissection. Follow-up PET-CT scan revealed recurrence in the left base of tongue and right level II lymph node. He was offered radiotherapy which he declined and opted to TORS and right neck dissection. Follow-up PET-CT scan showed recurrence in left RPN for which he underwent salvage concurrent chemoradiotherapy to 70 Gy. *Results*. After a followup of 9 months from the date of salvage chemoradiotherapy completion, the patient is with no evidence of disease. *Conclusions*. TORS followed by adjuvant radiotherapy seems reasonable in the context of squamous cell carcinoma of the head and neck due to the odds of RPN involvement. Further reports are warranted to optimize post-TORS adjuvant treatment.

## 1. Introduction

Transoral robotic surgery (TORS) has increased in popularity in a variety of different indications. In 2006, O'Malley et al. began the introduction and application of the Intuitive Surgical da Vinci robot to head and neck surgery [1]. Hurtuk et al. reported on 64 patients with oropharyngeal carcinoma. Out of 64 patients, 50% of stages I and II patients were spared adjuvant radiation therapy (RT) or combined chemoradiation (chemo-RT) while 34% of stages III and IV patients were spared chemotherapy [2]. Obviously, most patients treated with definitive RT or chemo-RT are spared TORS and spared neck dissection (ND). However TORS does not address the nodal disease of the neck which may require a second surgical procedure. Furthermore, TORS and neck dissection (ND) do not address the retropharyngeal lymph nodes (RPN). Thus while TORS can be an excellent approach, it usually requires multiple procedures and leaves a key area unaddressed. In the current report we highlight the impact of lack of adjuvant radiotherapy (RT) on RPN recurrence following TORS.

## 2. Case Report

A 58-year-old Caucasian male presented with painless solitary left-sided upper neck mass of 2-month duration. He reports smoking one pack per day for 30 years but denies alcohol or drug abuse. After comprehensive physical examination, which was unremarkable, fine needle aspiration (FNA) confirmed squamous cell carcinoma (SCC). A PET-CT scan showed single FDG avid 3.5 cm left cervical LN at level II.

## 3. Management

### 3.1. Surgery

The patient was presented at our institutional head and neck tumor conference and the consensus was to proceed with multiple targeted biopsies from the base of tongue (BOT) and bilateral tonsillectomy all of which were negative for malignancy. The patient was offered RT as a definitive management for metastasis of unknown primary (MUP) clinical stage T0N2aM0, stage IVA, but he opted to left ND. Pathology revealed one positive LN for SCC, at level II, out of fifty six LNs. It measured 4.5 cm in the greatest dimension with no extracapsular extension. The pathologic staging was MUP stage IVA (pT0pN2aM0).

### 3.2. Followup

**Figure 1:**
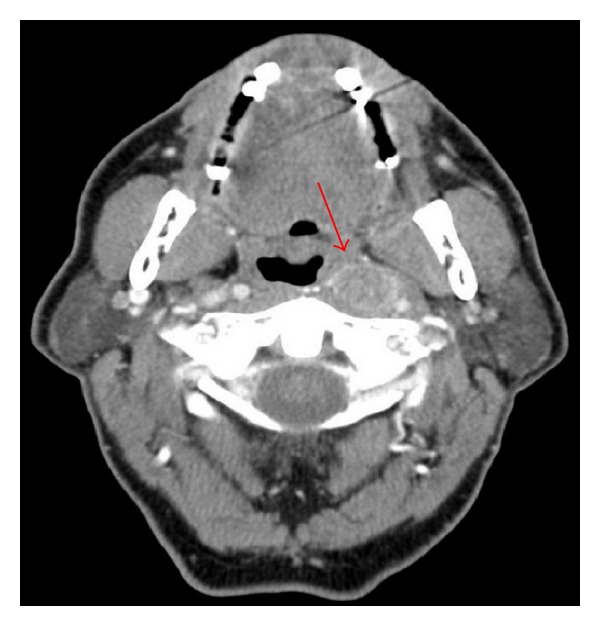
The solitary left lateral retropharyngeal lymph node involvement.

PET-CT scan, 6 months later, showed interval development of an intense hypermetabolic focus in the region of the left BOT and right level II LN. He underwent incisional biopsy of the left BOT mass, which was positive for SCC and HPV/p16. The patient was offered RT which he declined and opted to TORS and right ND, which revealed SCC of the left BOT, tumor measured 1.7 cm (pT1) with negative margins, and all the 29 LN were negative (pN0). He was pathologically staged as BOT, pT1pN0 M0, stage I. It is not clear whether the patient developed a second primary in the form of squamous cell carcinoma of the BOT or the primary of the initial undiagnosed MUP has emerged. Subsequently, he was offered adjuvant RT but he persistently declined. The patient did very well until a follow-up PET-CT scan (18 months later) showed a solitary 1.5 × 1.3 × 2.5 cm (Figure 1) markedly hypermetabolic left RPN with SUV of 9. He underwent a CT-guided FNA with pathology positive for SCC.

### 3.3. Radiotherapy

**Figure 2:**
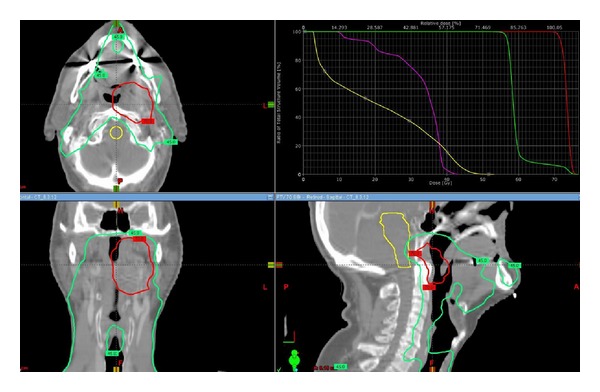
The isodose curves of RT treatment plan: axial, sagittal, and coronal views of PTVs 70 and 54 Gy.

The patient was represented at our institutional multidisciplinary head and neck tumor conference and the consensus was to proceed with salvage concurrent chemo-RT. The patient underwent CT simulation and IMRT based treatment. Two PTVs with dose painting were designed. The high dose region included the grossly enlarged FDG avid left RPN which received 70 Gy in 33 fractions. The lower dose for elective regions included the contralateral RPN, BOT, lymphatic in transit, and bilateral neck; all received 54 Gy in 33 fractions (Figures 2(a)–2(c)). He received concurrent chemotherapy in the form of cisplatin 100 mg/m^2^ (days 1, 22, 43). He experienced the expected acute RT related toxicity in the form of grade ≤2 (mucositis, dysgeusia, dysphagia, xerostomia, and dermatitis). After a followup of 9 months from the date of salvage chemo-RT completion, the patient is with no evidence of disease.

## 4. Discussion

TORS represents a shift from the conventional treatment paradigm on multiple levels. It does not require a mandibulotomy, mandibular swing, or tracheotomy for airway protection. Avoiding these surgical maneuvers provides patients with a far less morbid procedure [1–3]. Although the initial studies of TORS were focused on safety and efficacy, data regarding long-term oncologic results and functional outcomes are now available. Weinstein et al. reported on 47 patients with oropharyngeal SCC [3]. All patients underwent TORS + ND, 57% underwent post-TORS chemo-RT, 28% underwent post-TORS RT alone, and 2% underwent post-TORS chemotherapy alone. With a mean followup of 27 months, the local, regional, and distant control rates were 98%, 96%, and 91%, respectively. The 2-year actuarial overall and disease specific survival rates were 79% and 90%, respectively. Additionally, they reported 2.4% incidence of PEG dependence at 2 years, which is comparable to the current chemo-IMRT induced PEG dependence rates. Similar results have been reported by other investigators [1–7].

The primary drainage of the oropharynx is to the neck nodes (mainly level II) and to the lateral RPN. RPN are located in the retropharyngeal and parapharyngeal space that is closely related to cranial nerves IX through XII, the internal jugular vein, and the internal carotid artery at the base of skull which make them inaccessible surgically. Metastases to the RPN are most commonly associated with cancers of the nasopharynx, oropharynx, and pharyngeal wall. Notably, these metastases occur primarily along the lateral RPN chains. Involvement of the medial chain is extremely rare [8, 9]. 

The dismal clinical impact of RPN metastases has been reported in the literature. McLaughlin et al. reported on 774 patients with SCCHN. They found that the number of cervical nodal groups involved was the most significant factor (*P* < .0001) relating to the incidence of RPN involvement. The rates of neck relapse (40% at 5 years) and distant metastasis were significantly higher in patients with RPN involvement, and the rates of 5-year disease-free survival and absolute survival were significantly lower. They concluded that RPN involvement is a strong predictor of poor prognosis [10].

RT is used as adjuvant therapy, ±chemotherapy as dictated by the surgical pathology. Due to the rarity of reports that addressed the RPN involvement after TORS, there is no universal consensus on the management of this situation but traditionally salvage RT ± chemotherapy would be recommended. To the best of our knowledge there is no data to support the routine use of adjuvant RT after TORS especially with favorable pathological findings. However, due to the odds of RPN involvement in the context of oropharyngeal tumors, we believe that post-TORS adjuvant RT would be wise, as it comprehensively covers all areas at risk. In cases with adverse prognostic features (positive margins and extra capsular extension) concurrent chemo-RT should be offered [11, 12].

In the current report, salvage chemo-RT may offer a successful regional control for the RPN with acceptable toxicities. This case report is particularly important because it is unlikely that a prospective trial will be performed in this patient population. As there is little in the literature to guide treatment, we treated this patient in a similar fashion to salvage treatment strategy to SCCHN with complete success thus far despite the short followup. The real risks of local, regional nodal relapse or metastatic potential after TORS are unknown. Therefore, the appropriate areas to receive higher or lower doses, including nodal levels, are unclear. 

Another issue that must be considered in an era of depleting health resources is the costeffectiveness of the interventions. Investigators have reported that the costs of multimodality approach (i.e., TORS, ND, RT ± chemotherapy) were 10 times the cost of treatment with chemo-RT alone for operable tumors of the oropharynx. The majority of cost was related to inpatient and outpatient care, rather than surgical procedure [13–15]. Thus, while TORS can be an excellent approach, there are important issues that need to be addressed. This becomes a real discussion with the patient to truly present the pros and cons of all treatment approaches, so that the patients can make the right decision for them.

## 5. Conclusion

TORS followed by adjuvant RT seems reasonable in the context of BOT of the head and neck due to the odds of lateral RPN involvement. Further reports are warranted to optimize post-TORS adjuvant treatment.
